# Impending disaster: rise of the epsilon variant in Pakistan and the way forward

**DOI:** 10.1093/inthealth/ihab068

**Published:** 2021-10-07

**Authors:** Alina Moin, Shajie Ur Rehman Usmani, Hashim Khan

**Affiliations:** Dow University of Health Sciences, Baba e Urdu Road, Karachi 74200, Pakistan; Dow University of Health Sciences, Baba e Urdu Road, Karachi 74200, Pakistan; Dow University of Health Sciences, Baba e Urdu Road, Karachi 74200, Pakistan

**Keywords:** COVID-19 pandemic, developing countries, epsilon variant, Pakistan, strategic planning

## Abstract

The constantly mutating severe acute respiratory syndrome coronavirus 2 (SARS-COV-2) does not appear to be slowing down any time soon. All countries, particularly developing countries, must adapt and strategically plan their way of life around the pandemic, while doing everything possible to keep the mortality rate and spread of the newly emerging variants as low as possible, in order to avoid a further blow to the economy and way of life. Pakistan is one such developing country that is currently battling the dangerous delta strain of SARS-COV-2 with limited resources and has recently seen the emergence of an equally transmissible and highly infectious epsilon strain. This is a concerning situation considering that Pakistan's already overburdened health system and faltering economy cannot withstand another dangerous SARS-COV-2 variant attack. This article highlights some strategies for the country to fortify its defences to prevent the epsilon variant from spreading before it is too late, and emphasises that while identifying potential immune evasion mechanisms in SARS-COV-2 variants is critical in the fight against COVID-19, it is also critical to develop methods of efficient and cost-effective detection to identify an early outbreak and then vigilantly and systematically plan area lockdowns before any hope of conquering this pandemic is lost.

The COVID-19 pandemic has wreaked havoc and the constant mutations of severe acute respiratory syndrome coronavirus 2 (SARS-COV-2) have led to several questions, one of which is: Will lower-income countries be able to put up a fight against these freshly mutated variants? Similar concerns have arisen in Pakistan, where health authorities have issued another threat alert following the detection of five cases of the Californian ‘epsilon’ strain of SARS-COV-2 in Lahore.^1^ It is indeed a worrying situation considering that this variant has been classified by the WHO under ‘alerts for further monitoring’ due to its high transmissibility.

With the already prevalent delta strain of SARS-COV-2 spreading like wildfire, as of 15 August 2021, Pakistan had seen 3669 new cases, increasing the total number of active cases to 88 588 and 72 new deaths.^2^ Pakistan's overburdened and heavily saturated health infrastructure and a failing economy have made the country extremely vulnerable to a disastrous situation, similar to that faced by India as a result of the same variant. Hence, tackling the emerging variants of SARS-COV-2 now requires more thorough strategic planning instead of conventional approaches to limit transmission.

In a low-income country like Pakistan, mass lockdowns limiting the spread of COVID-19 are not feasible as they have contributed to growing unemployment and poverty, worsening an already downfallen economy. A wiser approach would be to impose micro-area ‘smart’ lockdowns combined with a stricter check on standard operating procedures, including social distancing and the use of masks. This will not only prevent further spread of the delta and epsilon variants, but also mitigate the impact on the livelihoods of the population and lessen the gravity of the negative impact on the economy. This methodology has been reinforced by the commendable Taiwanese effort of effectively tackling an epsilon variant outbreak in a hospital by imposing rigorous control measures and estimating transmission parameters to prevent the risk of future cases.^3^

Another important step would be surveillance and early detection of the epsilon variant and other emerging variants, which can ideally be accomplished by rigorous contact-tracing and by developing an efficient yet cost-effective method for detection. A study demonstrated a promising method for achieving this goal by using a simple RT-PCR assay rather than genetic sequencing, which is both time and money consuming.^4^ Moreover, it is necessary to identify potential immune evasion mechanisms displayed by various SARS-COV-2 variants, including the epsilon variant, in order to be able to target them therapeutically.

Perhaps the top tier measure in the fight against COVID-19 would be the development of a vaccine that can combat all variants. In this regard, Al Saba et al. proposed a propitious design of a multi-epitope targeted vaccine against SAR-COV-2, which could prove to be a promising endeavour as such a vaccine will enable low-income countries, including Pakistan, to actively combat the newly emerging stubborn variants of SARS-COV-2. This effort, in turn, can lead to a drastic decrease in burden on their healthcare systems by lowering patient influx and hospitalisations.^5^

In conclusion, because of a much slower vaccination rate compared with the rest of the world and the rising threat of newer variants, it is vital that Pakistan, along with all vulnerable developing countries, steps up and strengthens its defence lines through implementation of the aforementioned measures, backed by strong nation-state cooperation, before we reach the brink of an impending collapse.

## Data Availability

All data are transparent.
